# Mechanochemical and solvent-free assembly of zirconium-based metal–organic frameworks†
†Electronic supplementary information (ESI) available: Selected PXRD, FTIR-ATR, BET, TGA, SEM and DLS data. See DOI: 10.1039/c5cc08972g



**DOI:** 10.1039/c5cc08972g

**Published:** 2015-12-23

**Authors:** Krunoslav Užarević, Timothy C. Wang, Su-Young Moon, Athena M. Fidelli, Joseph T. Hupp, Omar K. Farha, Tomislav Friščić

**Affiliations:** a Department of Chemistry , McGill University , 801 Sherbrooke St. W. , H3A 0B8 Montreal , Canada . Email: tomislav.friscic@mcgill.ca; b Institute Ruder Boskovic , Zagreb , Croatia; c Department of Chemistry and International Institute for Nanotechnology , Northwestern University , Evanston , IL 60208 , USA . Email: o-farha@northwestern.edu; d Department of Chemistry , Faculty of Science , King Abdulaziz University , Jeddah , Saudi Arabia

## Abstract

We develop the first mechanochemical and solvent-free routes for zirconium metal–organic frameworks, making the frameworks UiO-66 and UiO-66-NH_2_ accessible on the gram scale without strong acids, high temperatures or excess reactants. The frameworks form either by milling, or spontaneous self-assembly by simply exposing solid mixtures of reactants to organic vapour. The generated frameworks exhibit high porosity and catalytic activity in the hydrolysis of model nerve agents, on par with their solvothermally generated counterparts.

## Abstract 2

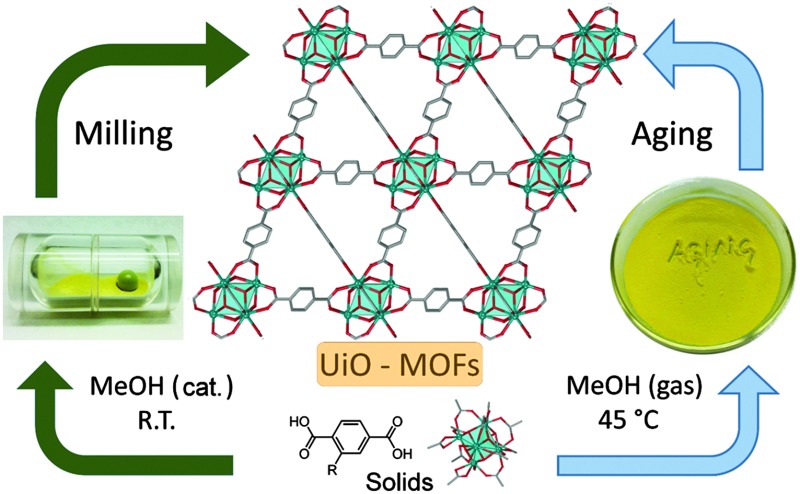
Mechanochemistry and accelerated aging are new routes to zirconium metal–organic frameworks, yielding UiO-66 and catalytically active UiO-66-NH_2_ accessible on the gram scale through mild solid-state self-assembly, without strong acids, high temperatures or excess reactants.

Metal–organic frameworks (MOFs)^1^ are one of the most active areas of materials science, with proof-of-concept applications in gas storage and separation,^2,3^ catalysis,^4^ chemical defense,^5^ sensing,^6^ light harvesting^7^ and more. The growing impact of MOFs is reflected by their recent commercialisation, which made a small number of MOFs based on Mg, Al, Fe, Cu and Zn commercially available on the laboratory research scale.^8^ However, chemical stability to strong acids or bases, and retention of microporosity upon extended exposure to different atmospheres remain central problems for most MOFs, including those currently being manufactured commercially.^9^

MOFs based on carboxylate linkers and 12-coordinate cationic Zr_6_O_4_(OH)_4_^12+^ clusters (Fig. 1),^10^ exemplified by terephthalate-based UiO-66^11^ and derivatives, have great potential as catalysts,^12^ given a high concentration of well-dispersed metal-based nodes, exceptional aqueous stability over a range of pH values, high surface areas, and tunable chemical structures.^13^ Despite continuous efforts and improvements,^14^ however, the syntheses of UiO-66 and analogues remain encumbered by challenging procedures involving aggressive reagents.^11a,15^ So far, the most reliable syntheses of UiO-66 and related MOFs rely on hydrochloric acid in hot organic solvents, achieving reproducibility at the expense of structural defects.^16^ Thus, the exploitation and potential commercialisation of excellent material properties of UiO-66 and analogues remain hindered by adverse synthesis.

**Fig. 1:**
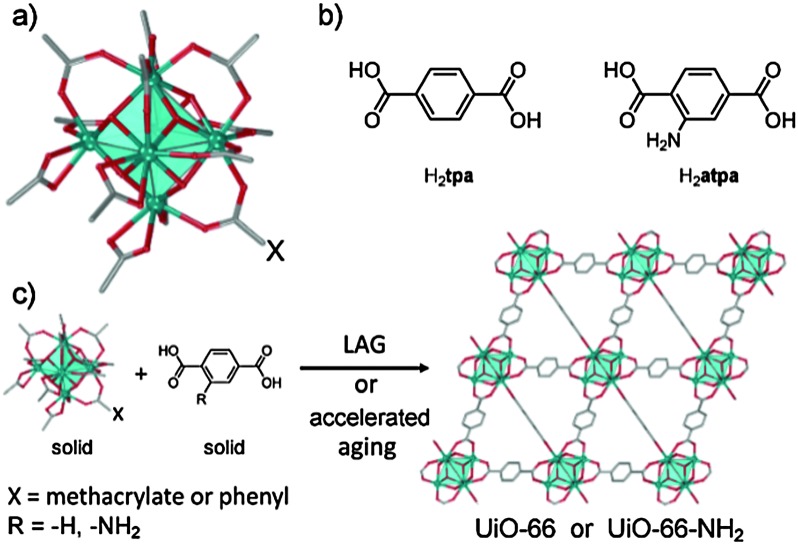
(a) A carboxylate-capped Zr_6_O_4_(OH)_4_^12+^ cluster; (b) terephthalic and 2-aminoterephthalic acids; (c) herein developed syntheses of UiO-type MOFs.

Mechanochemistry, *i.e.* reactivity induced or sustained by mechanical force,^17^ has been recently introduced as an alternative to conventional MOF syntheses.^18^ Mechanochemical techniques, such as liquid-assisted grinding (LAG),^19^ can enable the synthesis of MOFs^20^ without bulk solvents, aggressive conditions and/or corrosive reagents frequently employed in solution syntheses.^21^ Importantly, varying the liquid additive (given as *η*, ratio of liquid volume to weight of reactants^22^) in LAG provides a unique opportunity to optimise and direct mechanochemical reactions without large modifications in the milling procedures. Recent applications of LAG enabled the synthesis of MOFs from poorly soluble metal oxides and discovery of novel phases.^23^

We now describe the first implementation of mechanochemistry and solvent-free ‘accelerated aging’ (AA)^24^ for the synthesis of zirconium-based MOFs, providing new, surprisingly simple routes to gram amounts of UiO-66 and its amino-analogue UiO-66-NH_2_^11,25^ without using bulk solvents, aggressive reagents or high temperatures.

The principal requirement for the synthesis of UiO-66 and related MOFs is the formation of Zr_6_O_4_(OH)_4_ nodes. We considered a mechanochemical strategy similar to that recently used for IRMOFs,^26^ *i.e.* reaction of a pre-assembled benzoate cluster Zr_6_O_4_(OH)_4_(C_6_H_5_CO_2_)_12_ (**1**) with terephthalic acid (H_2_ **tpa**, Fig. 1). Precursor **1** was readily obtained from zirconium propoxide, Zr(OPr)_4_ (see ESI†).^27^ Dry milling of **1** and H_2_ **tpa** in the correct stoichiometric ratio 1 : 6 did not lead to a chemical reaction, according to powder X-ray diffraction (PXRD) analysis of the reaction mixture after 90 min milling. Switching to LAG (*η* = 0.66 μL g^–1^ ^22^) with *N*,*N*-dimethylformamide (DMF) gave a new product exhibiting a broad PXRD feature consistent with the (111) reflection of UiO-66 (Fig. 2a–f, also see the ESI†). Product formation was accompanied by the complete disappearance of reactant X-ray reflections.

**Fig. 2:**
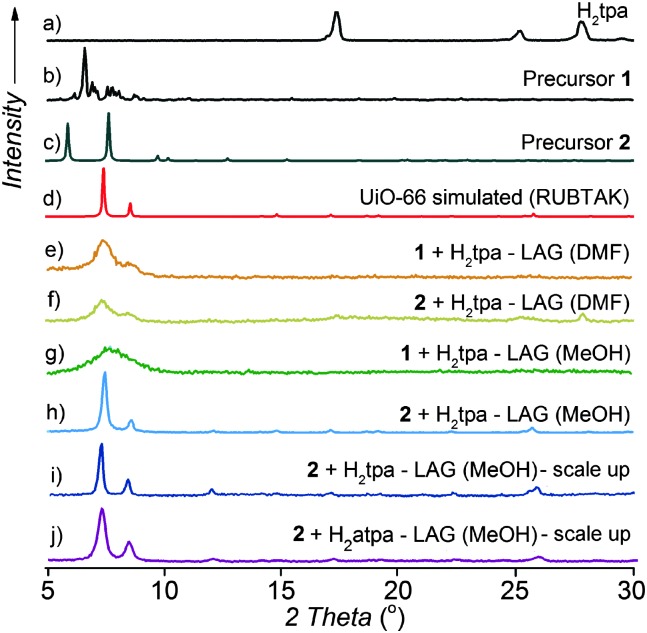
PXRD patterns for LAG syntheses of UiO-66 and UiO-66-NH_2_: (a) H_2_ **tpa**; (b) precursor **1**; (c) precursor **2**; (d) simulated for UiO-66 (CSD RUBTAK); (e) LAG of **1** and H_2_ **tpa** with DMF; (f) LAG of **2** and H_2_ **tpa** with DMF; (g) LAG of **1** and H_2_ **tpa** with MeOH; (h) LAG of **2** and H_2_ **tpa** with MeOH; (i) UiO-66 made on the 3 gram scale by LAG of **2** and H_2_ **tpa** with MeOH; (j) UiO-66-NH_2_ made on the 1.5 gram scale by LAG of **2** and H_2_ **atpa** with MeOH.

PXRD analysis suggests a successful exchange of benzoate ligands on **1**, leading to UiO-66 with poor crystallinity. An alternative precursor based on methacrylic acid, Zr_6_O_4_(OH)_4_(C_2_H_3_CO_2_)_12_ (**2**),^14c,28^ was used as well, without much improvement. At this point, we turned to varying the milling liquid. Switching from DMF to methanol (MeOH) led to little improvement in the crystallinity of product from **1** (Fig. 2g). However, LAG of **2** and H_2_ **tpa** with MeOH (*η* = 0.66 μL mg^–1^ ^22^) gave a product whose PXRD pattern exhibited sharp, well-defined reflections, with positions consistent with the UiO-66 structure (Fig. 2h). Washing was performed with MeOH only, allowing for the first time the complete exclusion of HCl and DMF from the synthesis of a UiO-type MOF. Using a Spex 8000 mill, the synthesis of UiO-66 by MeOH LAG was accomplished on the 3 g scale in 75 min (Fig. 2i and Fig. S1 and S2, ESI†). The BET surface area for the product, calculated from the N_2_ sorption isotherm at 77 K, was 1020 m^2^ g^–1^ (Table 1 and Fig. 3).^29^ Next, we targeted UiO-66-NH_2_, based on 2-aminoterephthalic acid (H_2_ **atpa**),^25^ recently established as an excellent catalyst for the hydrolytic degradation of nerve agent simulants.^30^ Based on the optimised mechanosynthesis of UiO-66, we milled **2** and H_2_ **atpa** in the presence of MeOH. After 90 min, PXRD revealed the formation of UiO-66-NH_2_, isostructural to UiO-66. Using a Spex 8000 mill, the mechanosynthesis of UiO-66-NH_2_ was performed on a 1.5 g scale in 45 min (Fig. 2j and Fig. S1–S3, ESI†). Based on a N_2_ isotherm at 77 K, the BET surface of UiO-66-NH_2_ after washing and activation was 945 m^2^ g^–1^ (Table 1 and Fig. 3a), consistent with the literature.^11,16a,25,31^

**Fig. 3:**
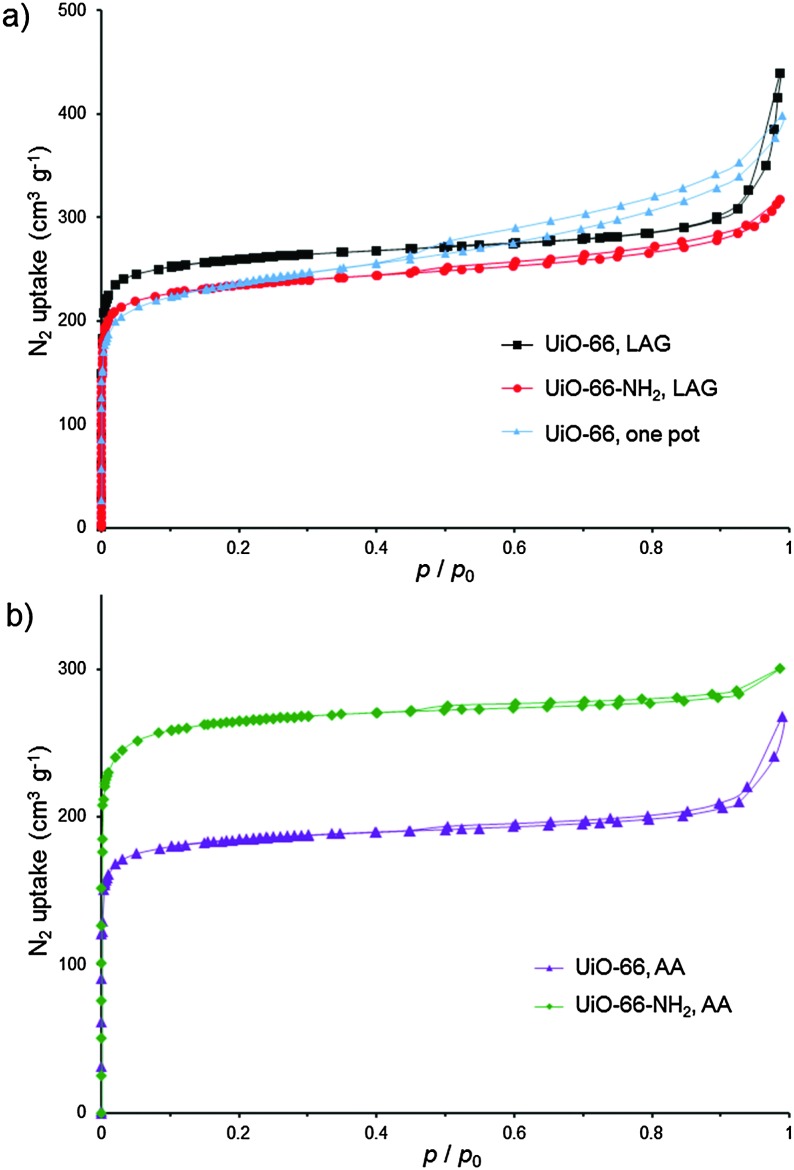
BET isotherms (N_2_, 77 K) for: (a) UiO-66 and UiO-66-NH_2_ from optimised LAG syntheses using MeOH, and UiO-66 made by a one-pot reaction from Zr(OPr)_4_; (b) UiO-66 and UiO-66-NH_2_ from AA in MeOH vapour at 45 °C.

**Table 1:** BET surface areas for mechanochemically made zirconium MOFs^*a*^ ^,^ ^*b*^

MOF	Precursor	BET surface area (m^2^ g^–1^)
UiO-66^29^	**2**	1020
UiO-66-NH_2_	**1**	925
UiO-66-NH_2_	**2**	945
UiO-66^29^	Zr(OPr)_4_ ^*c*^	890

^*a*^Details of activation are given in the ESI.

^*b*^All reactions were performed by LAG, using MeOH as the milling liquid.

^*c*^One-pot synthesis from Zr(OPr)_4_, acrylic acid and H_2_ **tpa** using MeOH as the grinding liquid.

UiO-66-NH_2_ was also accessible by LAG from **1**, giving a BET surface area of 925 m^2^ g^–1^ (Table 1 and Fig. S4, ESI†). The synthesis of UiO-66-NH_2_ involved only MeOH as the milling and washing liquid, again eliminating the need for DMF. Finally, we attempted a one-pot synthesis of UiO-66 directly from commercial zirconium propoxide, methacrylic acid and stoichiometric H_2_ **tpa**. After 90 min, one-pot milling in the presence of MeOH gave UiO-66 with a high surface area (Table 1, Fig. 3a and Fig. S5, ESI†), providing the first one-step route to UiO-66 from zirconium propoxide, an attractive replacement for ZrCl_4_ and ZrOCl_2_.

We next explored AA, an operationally simple technique that can achieve the solvent-free, low-energy synthesis of metal–organic materials^24^ by exposing a physical mixture of reactants to mild temperature and a suitable atmosphere. AA was previously applied to prepare salts, cocrystals, metal oxalate MOFs, zeolitic imidazolate frameworks and, recently, HKUST-1.^24^ Short grinding (<1 min in an agate mortar) of a physical mixture of **2** with H_2_ **tpa** or H_2_ **atpa**, followed by exposure to MeOH vapor at 45 °C led to the spontaneous assembly of UiO-66 and UiO-66-NH_2_, respectively (see ESI†). Throughout aging, the samples remained solid and the products were highly crystalline, as evidenced by PXRD, which revealed the complete disappearance of the reactants after 3 days (UiO-66-NH_2_) and 1 week (UiO-66) (Fig. S6 and S7, ESI†). MOFs, synthesised at the >1 g scale, exhibited high BET areas after activation (Table 2 and Fig. 3b).

**Table 2:** BET surface areas for MOFs made by accelerated aging in MeOH vapor^*a*^

MOF	Precursor	BET surface area (m^2^ g^–1^)
UiO-66^29^	**2**	730
UiO-66-NH_2_	**2**	1050
UiO-66-NH_2_	**1**	915

^*a*^Details of activation procedure are given in the ESI.

With the materials in hand, we examined the catalytic activity of UiO-66-NH_2_ made using LAG and AA. Both samples show high hydrolysis activity: 2.5 min and 2 min for an initial half-life for the degradation of the nerve agent simulant dimethyl 4-nitrophenyl phosphate (DMNP, Fig. 4), respectively. The catalytic activity of UiO-66-NH_2_ made using AA is comparable to that of solvothermally prepared materials (*t*_1/2_ = 1 min);^30^ the difference in half-life is attributed to a difference in the particle size. For mechanochemically made UiO-66-NH_2_, the average particle size was found to be below 100 nm by dynamic light scattering (DLS) in water (Fig. S8, ESI†). The slower reaction kinetics (*t*_1/2_ = 2.5 min) compared to those for UiO-66-NH_2_ made using AA is likely due to the rapid aggregation and precipitation of nanoparticles during the catalytic reaction. UiO-66 made using LAG or AA also exhibited high catalytic activity, on par with solvothermally made material.^5*a*^ The samples of UiO-66 and UiO-66-NH_2_ were characterised by scanning electron microscopy (SEM, see the ESI†) before and after evaluating the catalytic activity. Overall, the catalysis and porosity measurements show that zirconium MOFs made by LAG or AA have properties comparable to solvothermally made materials.

**Fig. 4:**
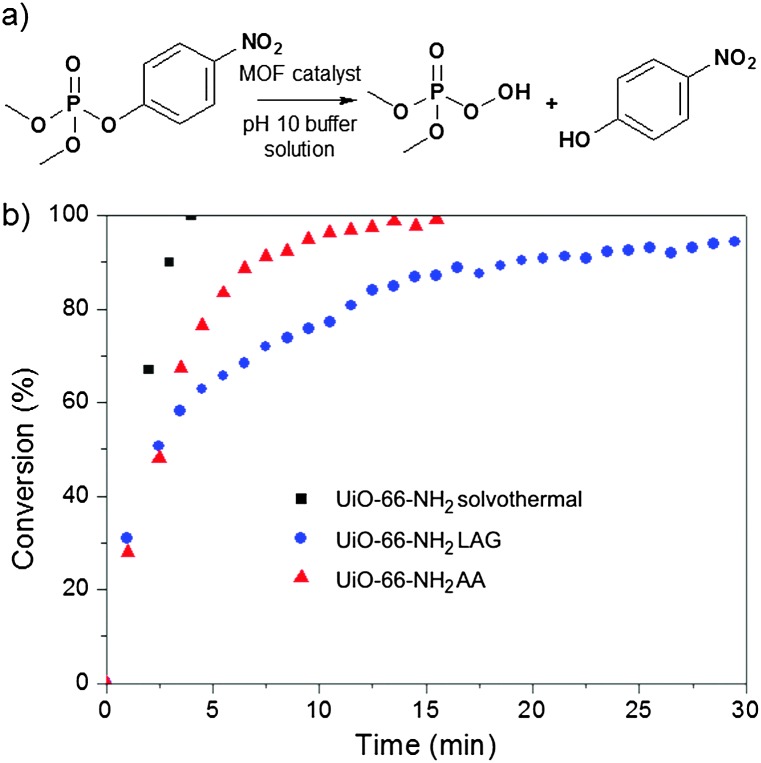
Hydrolysis of nerve agent simulant DMNP: (a) reaction; (b) hydrolysis profiles in the presence of UiO-66-NH_2_ made solvothermally (black), by LAG (blue) and AA (red).

We demonstrated that non-conventional synthetic approaches of mechanochemistry and accelerated aging enable a surprising simplification of procedures for making zirconium-based MOFs. The frameworks UiO-66 and UiO-66-NH_2_, noted for their outstanding stability and catalytic activity, can now be synthesised on the gram scale by a simple, rapid and room-temperature milling procedure which also allows using zirconium propoxide as the starting material.

Surprisingly, gram amounts of the microporous MOFs are obtainable by spontaneous assembly of the organic linker with a readily accessible carboxylate-capped zirconium cluster, simply by exposing a physical mixture of reactants to organic vapour. The herein presented techniques offer a route to highly porous, catalytically active UiO-frameworks under conditions resembling mild processes of small molecular self-assembly, in contrast to the conventional approaches that require acidic reagents (HCl, ZrCl_4_, ZrOCl_2_) under solvothermal conditions. These results have the potential to not only improve the wide accessibility of UiO-type MOFs but also to change the way microporous MOFs are synthesised in general.

We acknowledge the NSERC Discovery Grant for funding. KU is supported by the European Commission and the Croatian Ministry of Science, Education and Sports from the Marie Curie FP7-PEOPLE-2011-COFUND program NEWFELPRO, Grant Agreement No. 62. OKF and JTH acknowledge funding by the Army Research Office (project no. W911NF-13-1-0229). We acknowledge Tim Osborne-Jones (Spex SamplePrep LLC) for providing access to a Spex 8000 mill.
