# Supplementary material for: Mechanochemical and solvent-free assembly of zirconium-based metal–organic frameworks
Source: Chem Commun (Camb). 2015 Dec 23;52(10):2133–6. doi: 10.1039/c5cc08972g (PMC4767137; doi:10.1039/c5cc08972g)
Supplement: Supplementary file 1 [file CC-052-C5CC08972G-s001.pdf]

Electronic Supplementary Material (ESI) for ChemComm.
This journal is © The Royal Society of Chemistry 2015

# Mechanochemical and Solvent-free Assembly of Zirconium-Based Metal-organic Frameworks

# Supplementary Information

Krunoslav Užarević,[a] Timothy Wang,[b] Su-Young Moon,[b] Athena M. Fidelli,[a] Joseph T. Hupp,[b] Omar K. Farha[b,c,*], Tomislav Friščić[a,*]

[a]Department of Chemistry, McGill University; [b]Department of Chemistry and International Institute for Nanotechnology, Northwestern University; [c]Department of Chemistry, Faculty of Science, King Abdulaziz University, Jeddah, Saudi Arabia

**S1. Materials and Methods** S2
**S2. Synthetic procedures** S3
**S3. Selected characterization data for UiO-66 and UiO-66-NH$_2$ from LAG** S5
**Figure S1.** TGA/DSC analysis of UiO-66-NH2 and UiO-66 made by LAG. S5
**Figure S2.** ATR-FTIR spectra selected samples PXRD patterns for milling syntheses of UiO-66-NH$_2$ S5
**Figure S3.** PXRD patterns for selected samples of UiO-66-NH$_2$ S6
**Figure S4.** Nitrogen sorption isotherm at 77 K, for UiO-66-NH$_2$ made by LAG of **1** and H$_2$**atpa** S6
**Figure S5.** PXRD pattern for UiO-66 made by one-pot synthesis from Zr(OPr)$_4$ S7
**S4. Selected characterization data for UiO-66 and UiO-66-NH$_2$ from accelerated aging (AA)** S7
**Figure S6.** PXRD data for UiO-66 and UiO-66-NH$_2$ from accelerated aging in MeOH vapor (45 °C) S7
**Figure S7.** TGA/DSC analysis of UiO-66-NH2 and UiO-66 made by AA on gram scale S8
**Figure S8.** Dynamic Light Scattering (DLS) measurements S8
**S5. Scanning electron microscopy (SEM) characterization of UiO-66 and UiO-66-NH$_2$ before and after evaluation catalytic activity** S9
**Figure S9.** SEM images for UiO-66-NH$_2$ made by AA before and after evaluating catalytic activity S9
**Figure S10.** SEM images for UiO-66-NH$_2$ made by LAG before and after evaluating catalytic activity S10

## S1. Materials and methods

Terephthalic acid, 2-aminoterephthalic acid, methacrylic acid, benzoic acid, and zirconium(IV) isopropoxide were all purchased from Sigma Aldrich. Methanol (MeOH) and dimethylformamide (DMF) were purchased from ACP Chemicals. All chemicals were used without further purification.

**Infrared spectroscopy** Fourier-transform infrared attenuated total reflectance (FTIR-ATR) spectra were recorded on Bruker Vertex 70 spectrometer with RockSolidT$^{M}$ interferometer in the range of 4400-400 cm$^{-1}$ with 4cm$^{-1}$ step.

**Thermal (TGA/DSC) analysis**. Thermogravimetric analyses were performed on a Mettler-Toledo TGA DSC 1 Star$^{e}$ system thermobalance using alumina crucibles under air stream with the heating rate of 5-10 °C min$^{-1}$. The results were processed with the Mettler STARe 9.01 software.

**Powder X-ray diffraction (PXRD)**. X-ray powder diffraction data was collected on a Bruker D2 diffractometer, using nickel-filtered Cu$K\alpha$ radiation in Bragg-Brentano geometry ($U$= 30kV, $I$= 10 mA). Diffractograms were collected in the angular range 4°-40° ($2\theta$) with a step size of 0.05° and 0.65 seconds per step. Diffractograms for activated samples were recorded on a Rigaku ATXG diffractometer equipped with an 18 kW Cu-rotating anode, MLO monochromator, and a high-count-rate scintillation detector, over a range of 3°-50° (2θ), scanning speed of 4$^{o}$/min scanning speed.

**Activation of samples** was performed by soaking in MeOH for 6 h and exchanging the supernatant with fresh MeOH. After exchanging the supernatant three times, samples were dried under vacuum oven at 60 °C. The samples were then degassed under dynamic vacuum on a SmartVacPrep at 120 °C for 12 h or until the outgassing rate of ≤ 0.02 mm Hg min$^{-1}$ was reached. The nitrogen isotherms of the activated samples were then measured on a Tristar II, and the measurements were conducted at 77 K with liquid nitrogen bath.

**Scanning electron microscopy** (SEM) images and energy- dispersive X-ray spectroscopy (EDS) mapping were recorded on a Hitachi SU8030 SEM.

**Nuclear Magnetic Resonance** (NMR) spectra were collected on 400 MHz Agilent DD MR-400 at IMSERC (Integrated Molecular Structure Education and Research Center) of Northwestern University.

**Dynamic Light Scattering** (DLS) measurements of hydrodynamic radii were carried on a Malvern Zetasizer Nano-ZS (Malvern Instruments).

# S2. Synthetic procedures

### Synthesis of benzoate precursor (1), and methacrylate precursor (2)

Synthesis of **1** was performed according to literature.[1] Precursor **2** was prepared by reaction of methacrylic acid and $Zr(PrO)_4$ (70% solution in *n*-propanol). Methacrylic acid (1.4 mL) was added to 2 mL $Zr(PrO)_4$ solution, with addition of a drop of water. Reaction mixture was stirred for 10 minutes using magnetic stirrer in open flask and left to stand for one hour before the volume of the solution was reduced to 1/4 of a starting volume. The formed colorless solid was filtered under vacuum and washed once with 2 mL of *i*-propanol (yields 70-85 %, by increasing the reactants quantities it is possible to synthesize readily 50 g of **2**). Products, colorless crystalline materials were subjected to PXRD and FTIR-ATR analyses prior to further work.

### Milling synthesis of UiO-66

Optimization LAG reactions were performed in zirconia ($ZrO_2$) or PTFE (Teflon) 10 mL jars, using one 3.5 g zirconia ball and Retsch MM400 mixer mill operating at 30 Hz. Reactants (metal cluster:terephthalic acid in 1:6 molar ratio) were placed to the same part of the milling vessel and gently mixed, followed by the addition of denoted volume of DMF or methanol to the mixture (ca. 200 µL of liquid for 300 mg of solid reactants). Milling was stopped at designated times, to collect the PXRD data of the reaction mixture. The experiment was considered finished when no X-ray reflections of reactants were visible in the PXRD, which was usually after 90 or 180 minutes. After the milling was finished, the white powder was scraped off the jar walls, washed either with DMF or methanol, filtered, dried and analyzed by PXRD, FTIR-ATR and TGA.

| **1** (benzoate) | **2** (methacrylate) | $H_2$**tpa** | Liquid | Product weight (after washing) |
|---|---|---|---|---|
| | 175 mg (0.103 mmol) | 100 mg (0.6 mmol) | MeOH 200 µL | 124 mg |
| | 150 mg (0.09 mmol) | 90 mg (0.54 mmol) | DMF 160 µL | 119 mg |
| 130 mg (0.05 mmol) | | 53 mg (0.31 mmol) | MeOH 130 µL | 71 mg |
| 122 mg (0.045 mmol) | | 50 mg (0.27 mmol) | DMF 130 µL | 77 mg |

### Gram-scale milling synthesis of UiO-66

Gram-scale LAG synthesis of UiO-66 (2.85 g) was performed in a zirconia ($ZrO_2$) 50 mL vessel, using two 3.0 g zirconia balls and Spex 8000 mixer mill. Reactants (**2**:terephthalic acid in 1:6 molar ratio) were placed in the milling vessel and gently mixed, followed by the addition of 1.9 mL MeOH for 3.107 g of solid reactants. After 75 minutes of milling the white powder was collected from the walls of the vessel with a Teflon spatula, washed with MeOH, filtered and dried in vacuum oven for 1h at room temperature before analysis by PXRD, FTIR-ATR and TGA.

### Milling synthesis of UiO-66-$NH_2$

Optimization LAG reactions were performed in zirconia ($ZrO_2$) or PTFE (Teflon) 10 mL jars, using one 3.5 g zirconia ball and Retsch MM400 mixer mill operating at 30 Hz. Reactants (cluster:**2**-aminoterephthalic acid in 1:6 molar ratio) were placed to the same part of the milling vessel and gently mixed, and DMF or methanol were added to the mixture (ca. 200 µL of liquid for 300 mg of solid reactants). Milling was stopped at designated times, to collect the PXRD data of the reaction mixture. The experiment was considered to be finished when no traces of starting clusters were visible in the PXRD, which was usually after 90 or 135 minutes milling. After the milling was finished, the pale yellow powder was collected from the walls of the jar, washed with methanol, filtered, dried and analyzed by PXRD, FTIR-ATR and TGA.

| **1** (benzoate) | **2** (methacrylate) | $H_2$**atpa** | Liquid | Product weight (after washing) |
|---|---|---|---|---|
| | 145 mg (0.085 mmol) | 94 mg (0.51 mmol) | MeOH 160 µL | 131 mg |

| | | | | |
|---|---|---|---|---|
| | 145 mg (0.085 mmol) | 93 mg (0.51 mmol) | DMF 160 µL | 135 mg |
| 120 mg (0.045 mmol) | | 53 mg (0.29 mmol) | MeOH 120 µL | 67 mg |
| 100 mg (0.038 mmol) | | 45 mg (0.24 mmol) | DMF 100 µL | 62 mg |

**Gram-scale milling synthesis of UiO-66-$NH_2$**

UiO-66-$NH_2$ (1.34 g) was successfully isolated after 45 minutes of milling in a zirconia ($ZrO_2$) 50 mL vessel, using two 3.0 g zirconia balls and Spex 8000 mixer mill. Precursor **2** (1 g) was mixed with 0.64 g of 2-aminoterephthalic acid (molar ratio 0.59:3.53), followed by the addition of 800 µl of MeOH. The final mixture was washed twice with MeOH, filtered and dried under vacuum before subjected to PXRD, FTIR-ATR and TGA analysis.

**One-pot synthesis of UiO-66 from zirconium propoxide**

One-pot reaction was conducted by mixing 200 µl $Zr(PrO)_4$ (70 w% solution in *n*-PrOH) with 140 µL methacrylic acid in a 10 mL zirconia milling vessel. $H_2$**tpa** (72 mg) was added to the reaction, vessel sealed, and milling conducted for 90 minutes using a Retsch MM400 mill. PXRD analysis showed a broad feature at 7.5° 2θ (no $H_2$**tpa** was evident), so 100 µL of MeOH were added to the mixture and milling continued for further 90 min to increase product crystallinity. The product was washed with MeOH before analysis.

**Synthesis of UiO-66 and UiO-66-$NH_2$ by accelerated aging in MeOH vapor**

Reactants (**1** or **2**:dicarboxylic acid in 1:6 molar ratio) were briefly ground manually in an agate mortar. The mixture was then dispersed in a Petri dish, placed in a sealed chamber along a container of MeOH. Reaction mixture was left to stand at 45 °C without any further mixing. UiO-66 samples were synthesized by this method in 7-10 days, after which the reaction mixture was washed with MeOH and filtered. The reaction time for UiO-66-$NH_2$ was 3 days. Described procedure was successfully applied for the synthesis of 1.37 g of UiO-66 (from 1.2 g of **2** and 0.70 g of $H_2$**tpa**), and 1.49 g of UiO-66-$NH_2$ (from 1.2 g of **2** and 0.80 g of $H_2$**atpa**).

**Nerve agent simulant hydrolysis experiments**

Hydrolysis reactions were observed by *in situ* $^{31}P$ NMR at room temperature. A catalyst (F.W: 1664, 2.6 mg, 1.5 µmol) was loaded into a 1.5 dram vial and 1 mL of 0.4 M *N*-ethylmorpholine solution (0.05 mL *N*-ethylmorpholine, 0.9 mL DI water/0.1 mL $D_2O$) was added and then sonicated for 5 min to disperse homogeneously. 4 µL (25 µmol) of DMNP was added to mixture solution and swirled for 10 s. The reaction mixture was then transferred to an NMR tube and the spectrum was immediately measured; the first data point was collected at 150 s after the start of the reaction. The progress of the reaction was monitored with 1 min increments for 30 min (number of scans = 16, delay time = 28 s). The solvent was 10 % $D_2O/H_2O$. To measure reaction conversions at 1 min, a reaction mixture was prepared under identical conditions and filtered using a 200 nm syringe filter at 1 min, thereby stopping the reaction and permitting the degree of completion to be assessed by $^{31}P$ NMR.

## S3. Selected characterization data for UiO-66 and UiO-66-$NH_2$ from LAG

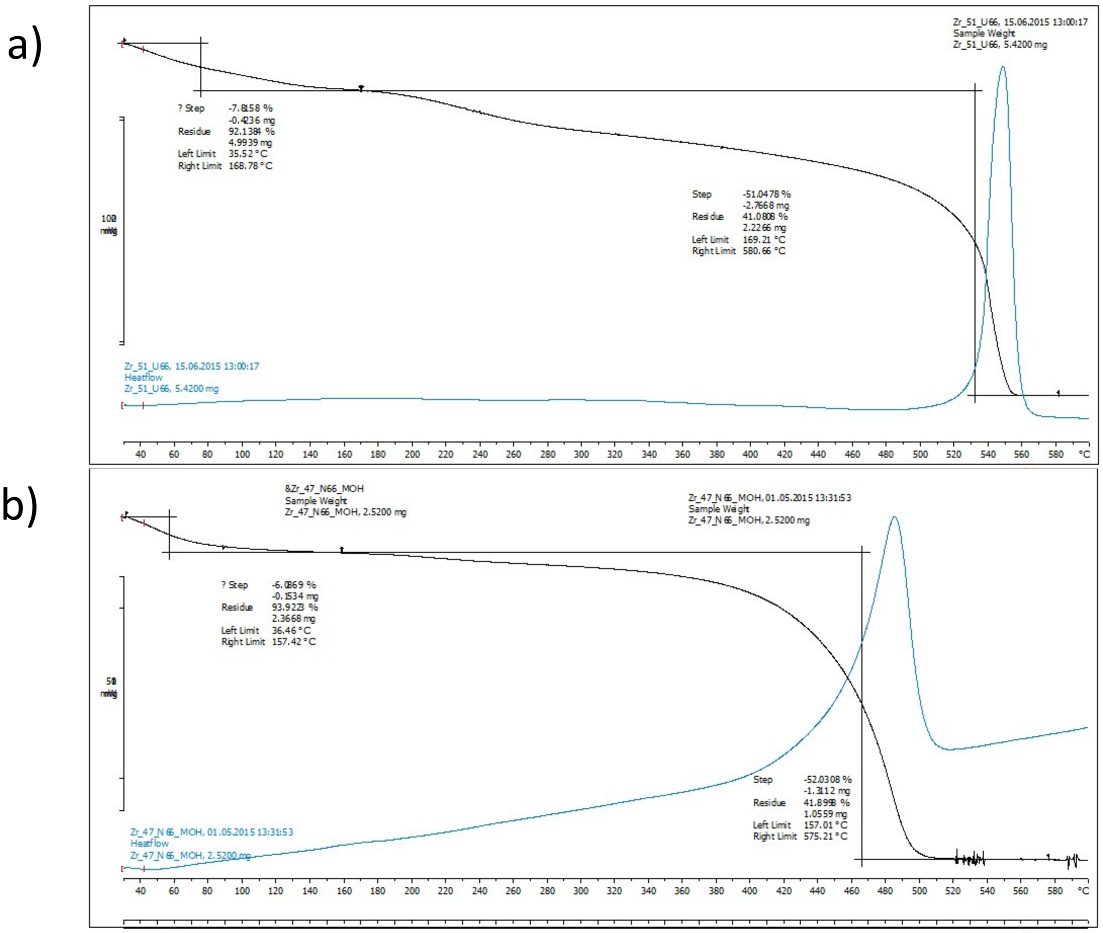


**Figure S1**. TGA/DSC curves (air, 30-600 °C, 10 °C/min) for: (a) UiO-66 and (b) UiO-66-$NH_2$ made by LAG using MeOH. TGA: black trace, DSC: blue trace.

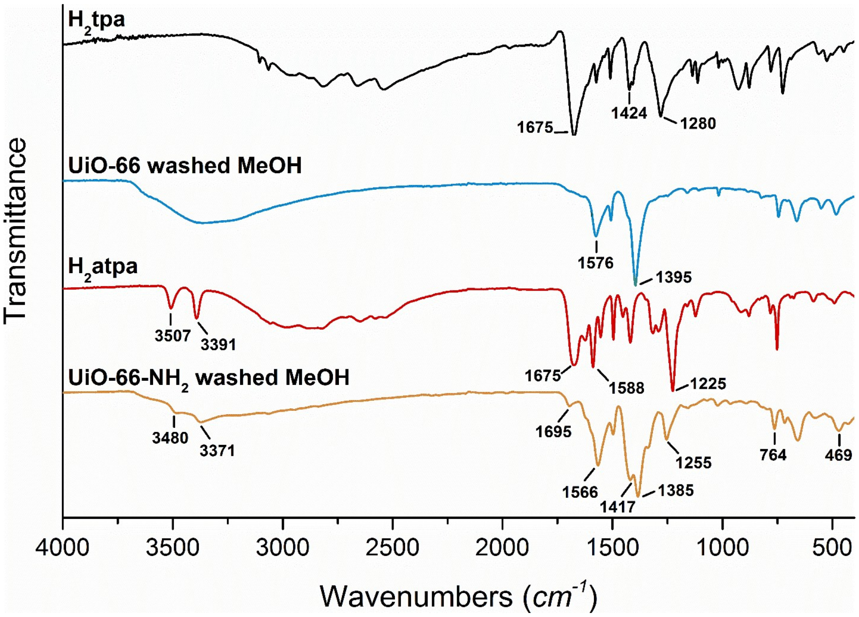


**Figure S2**. FTIR-ATR spectra for selected samples of UiO-66 and UiO-66-$NH_2$ made by milling, compared to free organic linkers.

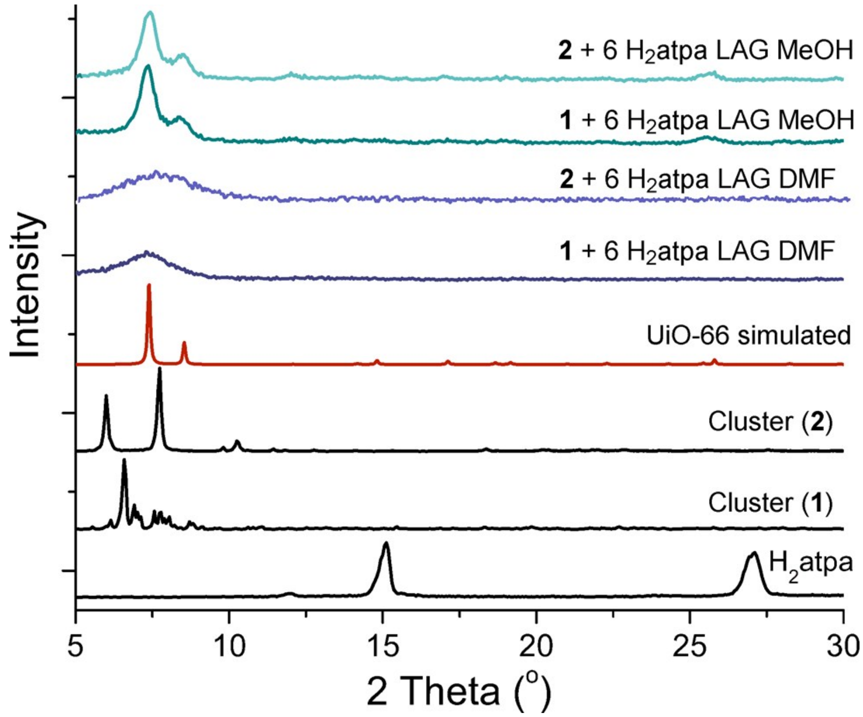


**Figure S3.** PXRD patterns for selected samples in milling synthesis of UiO-66-$NH_2$. The UiO-66 simulated pattern (red) corresponds to the CSD code RUBTAK.

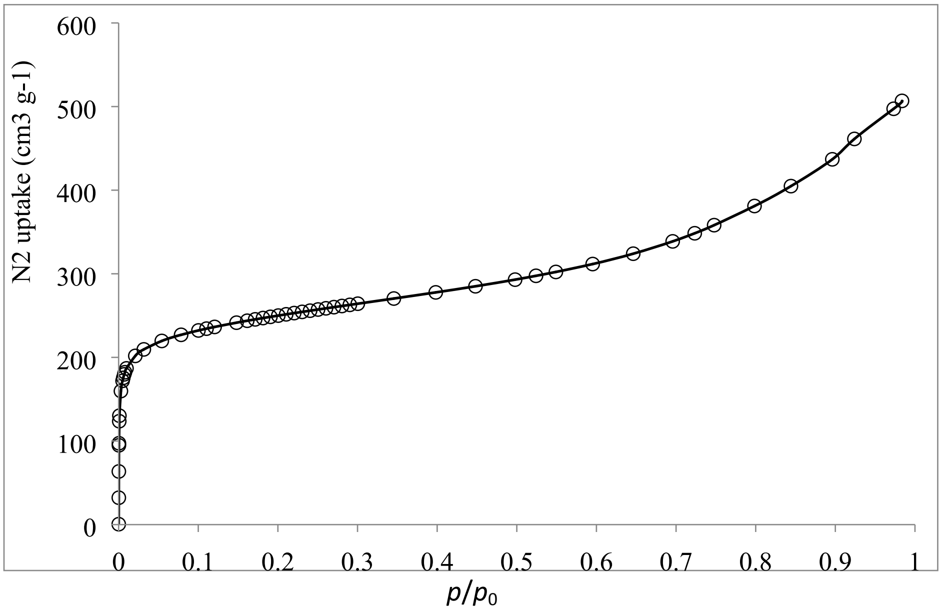


**Figure S4.** Nitrogen sorption isotherm at 77 K, measured for UiO-66-$NH_2$ made by LAG of **1** and $H_2$**atpa**, with MeOH grinding liquid.

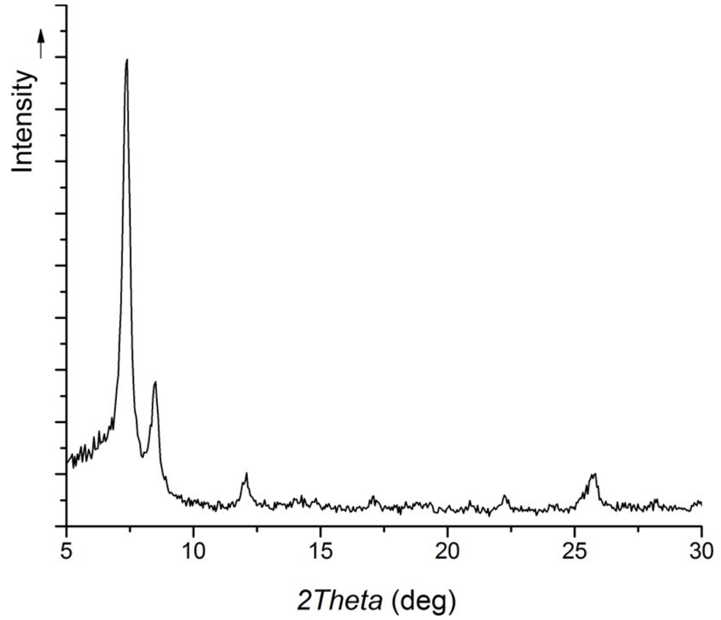


**Figure S5**. PXRD pattern for UiO-66 made by one-pot synthesis from $Zr(OPr)_4$.

## S4. Selected characterization data for UiO-66 and UiO-66-$NH_2$ from accelerated aging (AA)

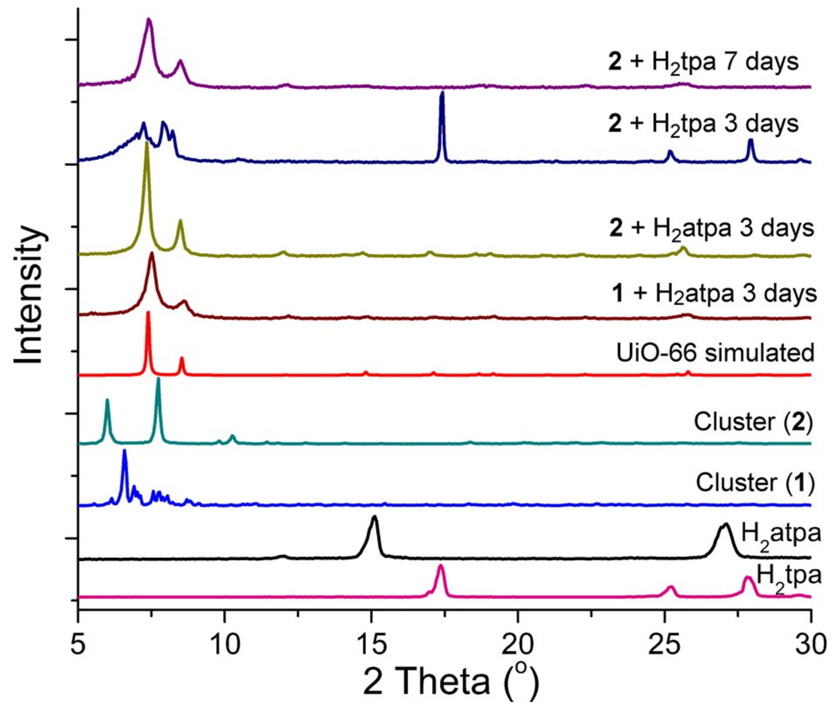


**Figure S6**. PXRD data for UiO-66 and UiO-66-$NH_2$ syntheses by accelerated aging in MeOH vapor (45 °C).

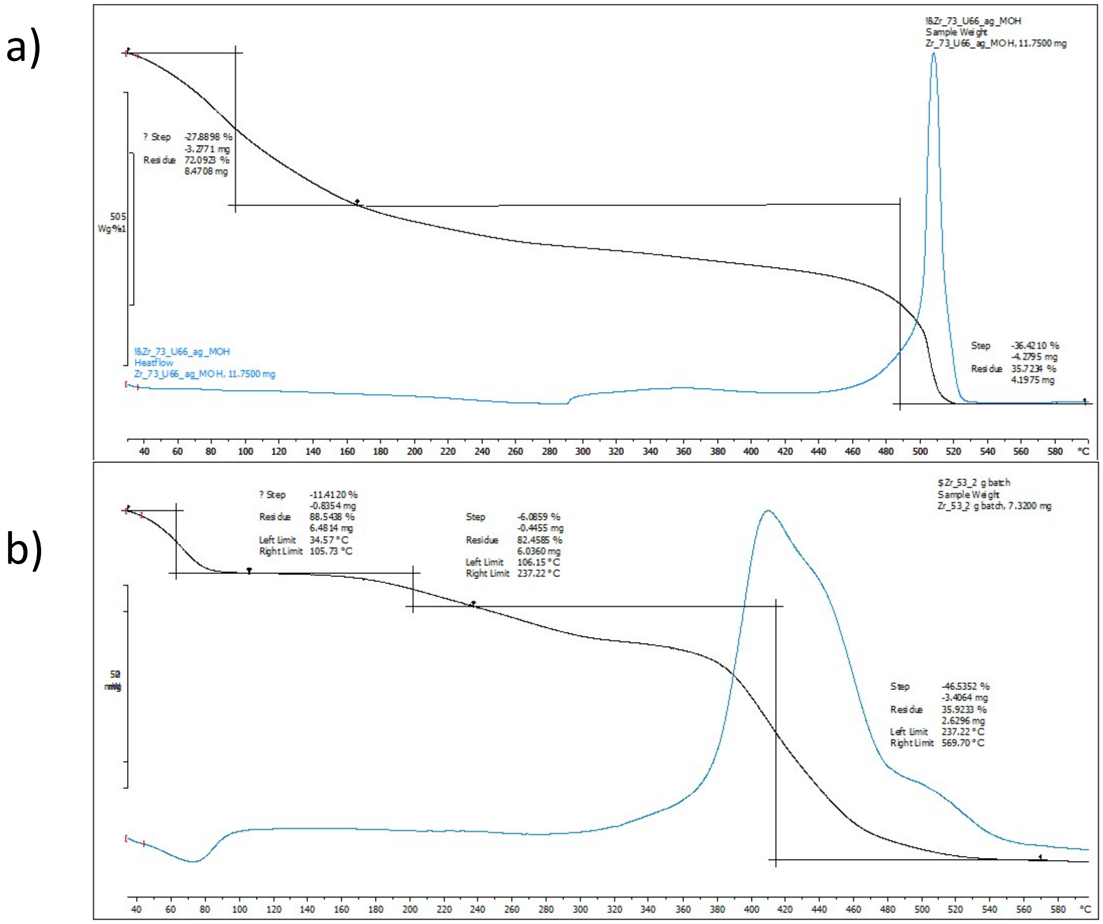


**Figure S7**. a) TGA/DSC (air, 30-600 °C, 10 °C/min) analysis of UiO-66 obtained by gram-scale accelerated aging in MeOH vapor at 45 °C and b) TGA/DSC analysis (air, 30-600 °C, 10 °C/min) for gram-scale synthesis of UiO-66-$NH_2$ by accelerated aging. TGA: black trace, DSC: blue trace.

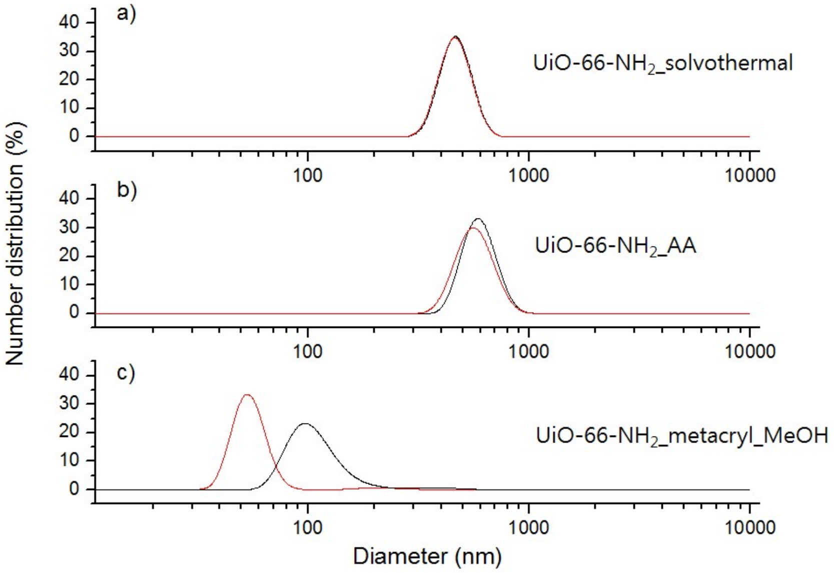


**Figure S8**. Dynamic Light Scattering (DLS) measurement in a dilute aqueous solution of a) UiO-66-$NH_2$-solvothermal, b) UiO-66-$NH_2$made by accelerated aging in MeOH vapor and c) UiO-66-$NH_2$ made by LAG using MeOH.

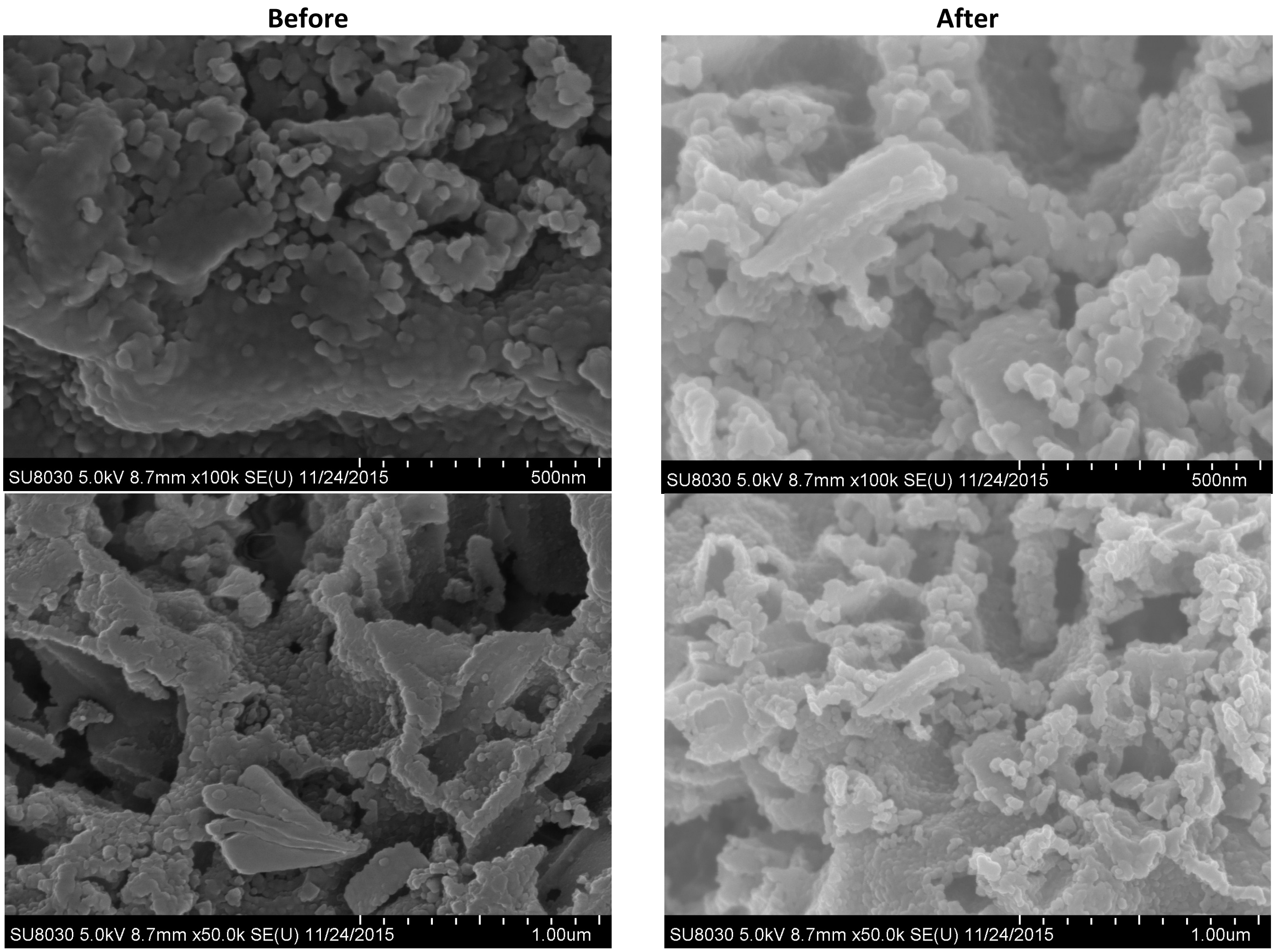


**Figure S9**. SEM images for a sample of UiO-66-$NH_2$ made by AA before (left) and after (right) evaluation of activity in catalytic hydrolytic degradation of the nerve gas simulant DMNP.

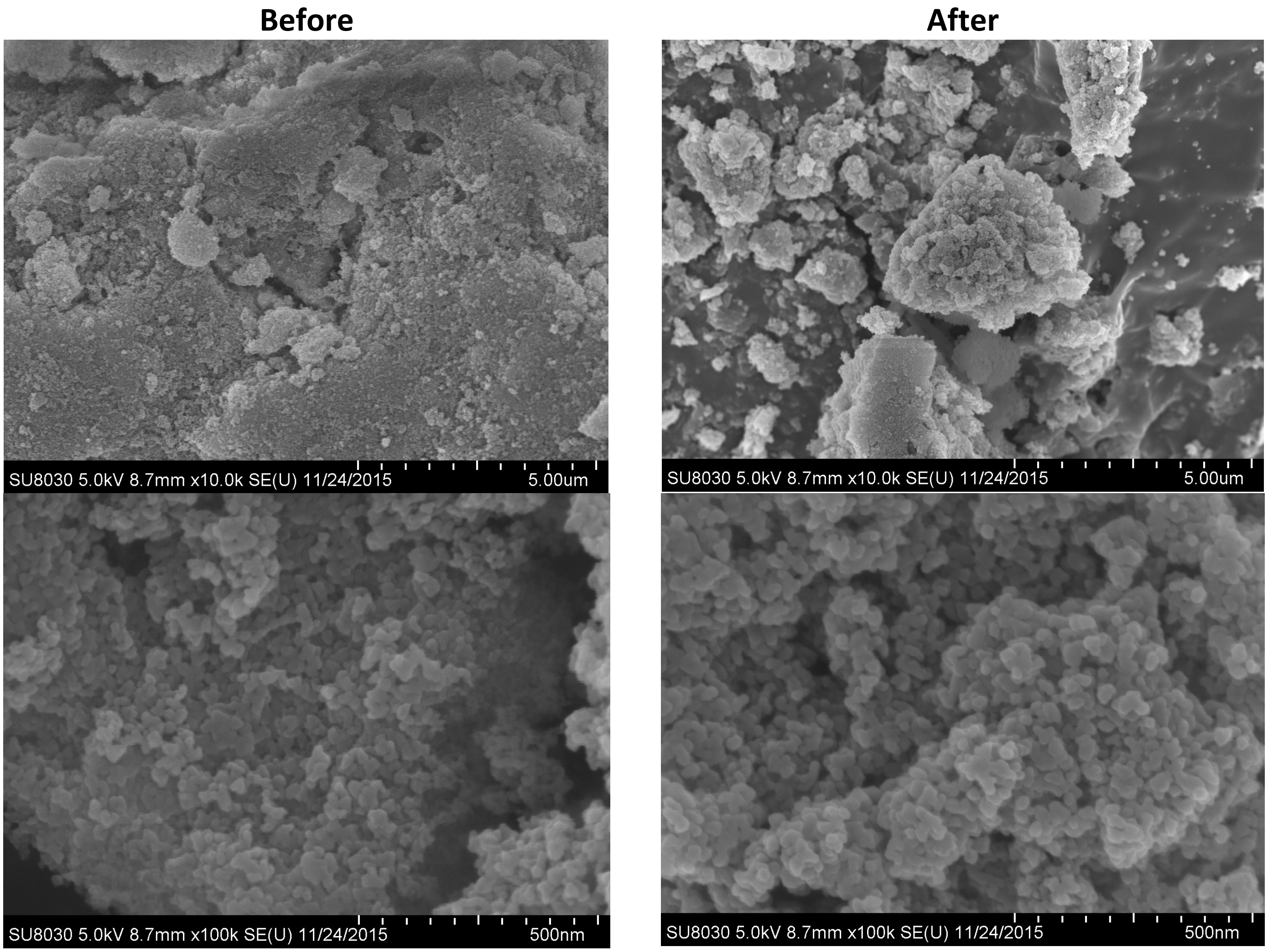


**Figure S10**. SEM images for a sample of UiO-66-$NH_2$ made by LAG before (left) and after (right) evaluation of activity in catalytic hydrolytic degradation of the nerve gas simulant DMNP.

---

[1] Kickelbick, G.; Wiede, P.; Schubert, U. *Inorg. Chim. Acta* **1999**, *287*, 1-7
